# Effects of Temperature-Control Admixtures on Shrinkage and Mechanical Properties of Fly Ash Concrete: Experiments and Modeling

**DOI:** 10.3390/ma18163757

**Published:** 2025-08-11

**Authors:** Yingda Zhang, Haiyang Li, Haojie Zhang, Xianliang Zhou, Ziyi Xu, Zihao Liu

**Affiliations:** 1 School of Architecture and Civil Engineering, Xihua University, Chengdu 610039, China; yingda.zhang@xhu.edu.cn (Y.Z.);; 2 Sichuan Provincial No. 12 Geological Team, Yibin 644002, China; 3 Key Laboratory of the Ministry of Education for Advanced Catalysis Materials, College of Chemistry and Materials Science, Zhejiang Normal University, Jinhua 321004, China; 4 School of Civil Engineering, Southwest Jiaotong University, Chengdu 610031, China; 5 Department of Architecture, Faculty of Environmental Engineering, The University of Kitakyushu, 1-1 Hibikino Wakamatsu, Kitakyushu, Fukuoka 8080135, Japan

**Keywords:** concrete shrinkage, temperature-control admixture, fly ash concrete, mechanical properties, shrinkage prediction models, early-age behavior

## Abstract

The mitigation of early-age shrinkage and thermal cracking remains a pressing challenge in mass concrete structures. This study introduces a novel temperature-control admixture (TCA), formulated with gel-forming inorganic compounds, designed to suppress internal temperature rise while improving the mechanical stability of fly ash concrete. Four concrete mixes with TCA dosages of 0, 0.05, 0.10, and 0.15% were experimentally evaluated under controlled environmental conditions. Results show that the optimal dosage of 0.10% achieved a 27.3% reduction in shrinkage and a 12.2% increase in compressive strength at 28 days compared to the control. Furthermore, existing shrinkage models (Eurocode 2, fib Model Code 2010, AS 3600, Bazant B4) consistently overestimated shrinkage by up to 294% due to their inability to capture TCA-induced modifications in hydration and moisture transport. To address this, a modified prediction model incorporating admixture and fly ash–dependent correction factors was proposed, reducing the mean prediction error to just 10% and achieving a coefficient of variation as low as 0.08. This work provides a semi-empirical modeling approach that captures the influence of microencapsulated TCAs on concrete shrinkage and offers useful insights for the design and optimization of advanced concrete systems.

## 1. Introduction

With the rapid advancement of construction technology and the increasing scale of structural dimensions towards ultra-high, ultra-long, and massive volumes, the challenges associated with early-age cracking in concrete have become increasingly prominent [1,2,3]. Among various factors, shrinkage-induced cracking, particularly thermal shrinkage cracking, poses significant threats to the durability and serviceability of concrete structures [4]. For example, early-age shrinkage has been identified as a major cause of cracking in concrete structures such as bridge decks. For example, transverse cracking observed in newly constructed bridge decks in Florida was partially attributed to shrinkage combined with restraint stresses, which led to durability issues and increased maintenance needs [5]. To mitigate shrinkage-induced cracking, engineering measures such as optimizing mix proportions, using shrinkage-reducing admixtures, and ensuring adequate curing have been widely adopted.

During the setting and hardening stages of concrete, substantial heat generation may occur [6,7]. If this heat is not dissipated efficiently, it results in significant temperature gradients between the interior and exterior of the structure, inducing non-uniform volume changes and thermal stresses [8,9]. When these stresses exceed the tensile strength of concrete, thermal cracking ensues [10]. Numerous engineering practices have demonstrated that incorporating temperature-control admixtures (TCAs) can effectively mitigate thermal cracking [11,12].

Temperature-control admixtures are designed to moderate the internal temperature rise of concrete by generating water-insoluble gels that fill pores, thus increasing the density and strength of the material [13]. These admixtures can lower the peak temperature or delay the temperature rise, thereby effectively controlling shrinkage. Although researchers have made considerable progress in understanding TCAs, the impact of different dosages on shrinkage behavior and mechanical properties requires further systematic experimental investigation to optimize mix designs.

To mitigate thermal cracking in mass concrete structures, several strategies have been developed, including optimizing mix proportions and incorporating various chemical admixtures and mineral additives [14,15,16]. The addition of appropriate admixtures can impart desirable properties to concrete, such as reduced heat of hydration, enhanced cracking resistance, and improved impermeability, to meet diverse engineering requirements [17,18]. Extensive studies have been conducted on the mechanisms of action, quality control, mix design, construction techniques, and performance evaluation of concrete incorporating admixtures [19,20].

A wide range of temperature-control admixtures have been explored, including titanium slag powder [21], sandstone powder [22], granite powder [23], and marble powder [24]. Commonly used chemical admixtures for thermal crack resistance include urea [25], sugars and their derivatives [26], phase-change materials (PCMs) [27], and microencapsulated systems [28]. For instance, urea (CO(NH_2_)_2_), a widely used organic compound, undergoes an endothermic reaction with water that lowers the environmental temperature during hydration, followed by an exothermic reaction that releases energy [29]. However, high dosages of urea can negatively impact concrete durability and strength, limiting its practical applications. Sugars and their derivatives, another common category of temperature-control admixtures, adsorb onto cement particles through negatively charged carboxyl groups during the early hydration stage, thereby inhibiting the formation of hydration products and reducing the heat evolution rate [30]. Phase-change materials (PCMs), which store or release latent heat during phase transitions, have also been proposed to control temperature fluctuations in concrete. Nevertheless, challenges such as poor encapsulation stability and the large volume fraction of lightweight aggregates required for PCM incorporation can result in reduced mechanical strength and thermal conductivity of cementitious materials, limiting their broader adoption [31]. Recently, microencapsulated temperature-control admixtures have been developed, offering effective temperature-control but significantly prolonging the induction period by suppressing hydration product formation [32].

Among the various TCAs, urea-based compounds, PCMs, and sugar-based bio-admixtures have been extensively explored. However, each of these types presents notable limitations. Urea exhibits excellent cooling effects but increases early-age porosity and decreases long-term strength, with potential durability concerns at high dosages. PCMs offer smooth temperature profiles via phase transitions but may lead to reduced mechanical performance due to volume replacement and poor integration. Sugar-based compounds can delay hydration but significantly prolong the setting time, potentially affecting construction schedules. In contrast, microencapsulated TCAs provide more controlled and sustained temperature regulation by gradually releasing active agents. As such, JX-E-type microencapsulated TCAs are used in this study. Despite the growing body of research on temperature-control admixtures, two key knowledge gaps remain. First, the majority of studies focus on the thermal behavior of TCAs but lack systematic evaluation of their influence on drying shrinkage and mechanical properties, particularly when used in conjunction with high-volume fly ash concrete. Second, existing shrinkage prediction models are not calibrated for concrete incorporating TCAs, leading to significant prediction errors in practical applications [33]. The internal temperature history during early-age curing plays a significant role in the development of shrinkage [34,35,36]. By altering the thermal evolution profile, temperature-control admixtures could potentially affect moisture migration, pore structure development, and subsequent shrinkage mechanisms [37,38].

Given that shrinkage is closely linked to both thermal and hydro histories of concrete, it is crucial to investigate whether the incorporation of temperature-control admixtures modifies the overall shrinkage behavior [39,40,41]. Furthermore, as existing shrinkage prediction models are calibrated based on conventional concrete without such admixtures, it is uncertain whether they can accurately predict the shrinkage performance of concrete incorporating temperature-control technologies. This highlights the necessity for experimental validation and potential model adjustments tailored to new-generation concrete materials.

Therefore, this study is driven by the hypothesis that there exists an optimal dosage of microencapsulated TCA that can achieve a balanced enhancement in both shrinkage mitigation and mechanical performance. To validate this hypothesis, a systematic experimental program was conducted using fly ash concrete incorporating varying TCA dosages (0, 0.05, 0.10, and 0.15%). First, the study aims to identify the critical dosage threshold beyond which the incorporation of TCA leads to a decline in mechanical strength. Moreover, the shrinkage behavior of each mixture is quantitatively assessed to reveal the relationship between TCA content and drying shrinkage [42]. Furthermore, by comparing experimental shrinkage results with predictions from Eurocode 2 [43], Model Code 2010 [44], AS 3600 [45], and the Bazant B4 model [46], this research evaluates the suitability of these models for modern fly ash concrete mixtures incorporating TCAs. Ultimately, a modified prediction model is proposed to better match the shrinkage behavior observed in experimental data, providing a theoretical foundation for the design and durability evaluation of concrete structures employing shrinkage-reducing technologies.

## 2. Experimental Program

### 2.1. Raw Materials and Mix Design

The cement used in this study is ordinary Portland cement (OPC) with a strength grade of 42.5 MPa, following the Chinese Standard GB 175-2023 [47]. The supplementary cementitious material is laboratory-grade fly ash (FA) produced by Longtan Company (Jinhua Xinsheng Zeolite Development Co., Ltd. (Jinhua, China)). The chemical compositions of fly ash and cement are presented in Table 1. Fine aggregate consists of river sand from Chengdu with a fineness modulus of 2.7. Coarse aggregate is continuously graded crushed stone with a particle size ranging from 5 to 20 mm. The temperature-control admixture used is JX-E type temperature-control and anti-cracking agent, supplied by Jinhua Xinsheng Zeolite Development Co., Ltd. The technical properties of the TCA are summarized in Table 2. Table 2 lists the technical indicators of the microencapsulated TCA used in this study. These values were provided by the manufacturer as part of the product’s quality certification. Although there is currently no standard regulation that specifies the required testing parameters for TCAs, the listed properties are commonly used in engineering practice to assess admixture performance. The TCA is added at dosages of 0, 0.05, 0.1, and 0.15% by weight of cement. A total of four different concrete mix designs are prepared, and the detailed mix proportions are presented in Table 3. The primary variable is the dosage of the temperature-control admixture. Group N refers to the normal concrete without admixture, while groups Y1, Y2, and Y3 correspond to concrete mixtures incorporating 0.05, 0.1, and 0.15% of the temperature-control admixture by weight of cement, respectively. These dosage levels were chosen based on both manufacturer recommendations and practical engineering considerations. The supplier of the JX-E admixture suggests a standard dosage of 0.10% by weight of cementitious materials. However, in real-world applications, the actual dosage is often adjusted—lowered to reduce cost or increased to enhance thermal control—depending on project-specific requirements. Therefore, the selected range of 0.05 to 0.15% encompasses the realistic field application spectrum and enables a comprehensive evaluation of performance variations across different dosages.

### 2.2. Mechanical Properties’ Test

**Figure 1:**
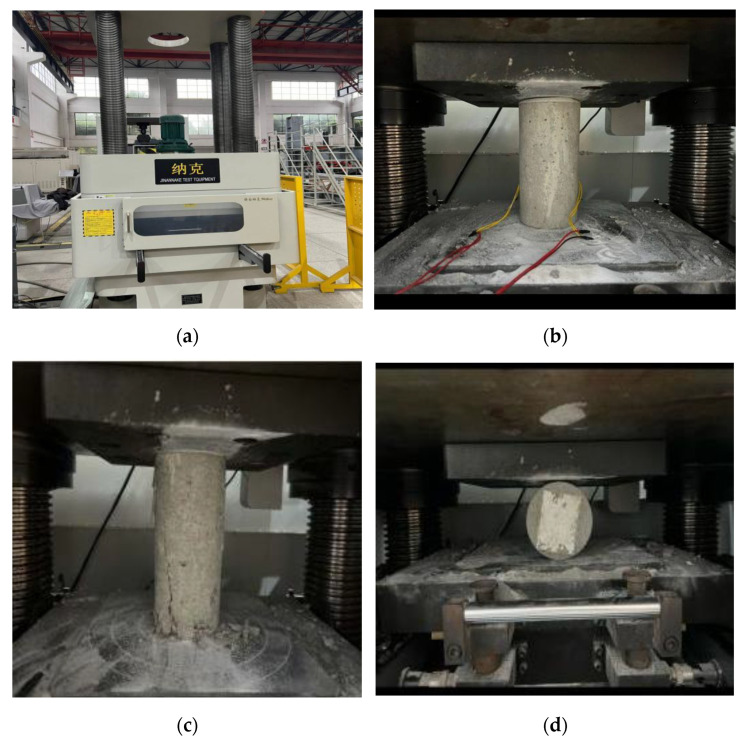
Computer-controlled electro-hydraulic servo testing machine: (**a**) external view; (**b**) elastic modulus test; (**c**) compressive strength test; (**d**) splitting tensile strength test.

The mechanical properties of concrete, including compressive strength, splitting tensile strength, and static elastic modulus, were evaluated using a Model YES-2000 universal testing machine from Jinan Nake Testing Equipment Co., Ltd. (Jinan, China). The testing equipment features load-closed loop control, constant stress control, and load-holding capabilities. The force measurement system employed a high-precision load sensor to ensure reliable accuracy. The specimen testing process is illustrated in Figure 1.

The compressive strength test was measured using cylindrical specimens with dimensions of 100 mm in diameter and 200 mm in height. A constant loading rate of 0.5 MPa/s was applied until failure. The compressive strength was calculated by dividing the maximum load at failure by the cross-sectional area of the specimen. Each data point represents the average of three specimens. The splitting tensile strength was measured using cylindrical specimens with dimensions of 100 mm in diameter and 200 mm in height. The specimens were subjected to a compressive load along their diameter at a loading rate of 0.05 MPa/s. The static elastic modulus was determined according to the standard procedures for concrete modulus testing. The specimens were loaded and unloaded cyclically within a predefined stress range (typically 0.3 to 0.4 times the ultimate compressive strength) to minimize plastic deformation. The slope of the linear portion of the stress–strain curve was used to calculate the elastic modulus. All mechanical property specimens were cured by full water immersion in a temperature-controlled curing room maintained at 20 ± 2 °C and approximately 100% relative humidity. The water used for curing was refreshed periodically to ensure stability. The specimens were removed from the curing tank and surface-dried prior to testing at 3, 7, or 28 days.

### 2.3. Shrinkage Test

**Figure 2:**
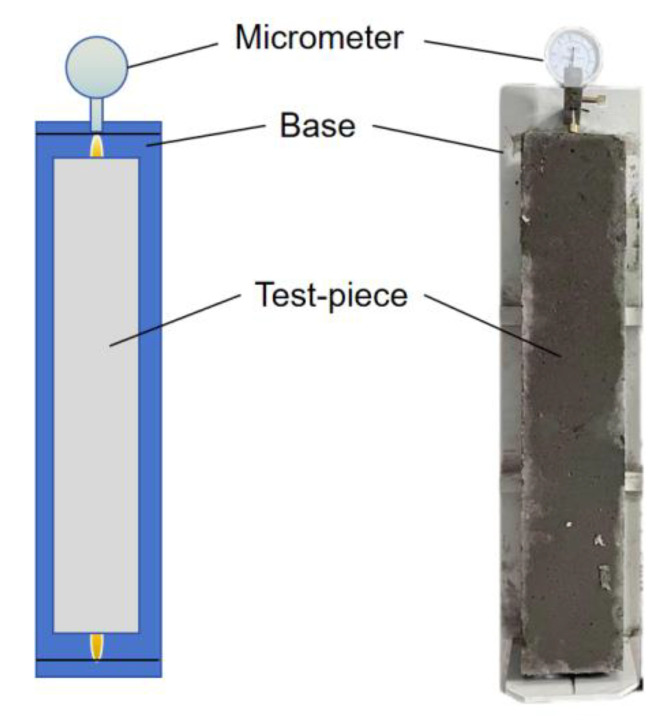
Concrete shrinkage testing device.

**Figure 3:**
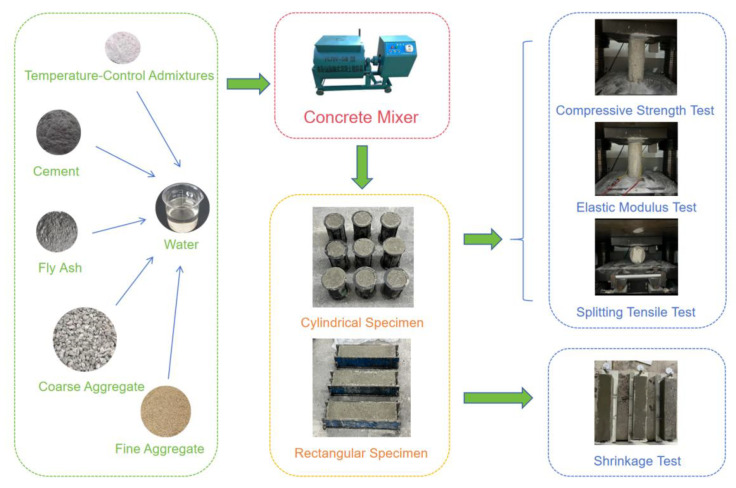
Technical flowchart of the experimental program.

The shrinkage behavior of concrete was evaluated by measuring the early-age autogenous shrinkage under controlled environmental conditions. The fresh concrete was cast into molds with dimensions of 515 mm × 100 mm × 100 mm. After initial setting, the specimens were carefully demolded and immediately placed into the early-age autogenous shrinkage testing device. Shrinkage specimens were not subjected to water curing. After 24 h and demolding, the specimens were stored in a temperature- and humidity-controlled chamber maintained at a temperature of 20 ± 2 °C and a relative humidity of 50 ± 5%. The shrinkage deformation was measured periodically throughout the curing process to monitor the time-dependent development of concrete shrinkage. Each mix group was tested with three specimens, and the average shrinkage value was recorded. The early-age concrete shrinkage testing device, as shown in Figure 2, consists of three main components: the concrete specimen, a support tray, and a micrometer gauge. The micrometer gauge was used to measure the change in specimen length with a precision of 1 μm. The setup allows continuous monitoring of the shrinkage without restraint, ensuring that the measurements reflected the intrinsic autogenous deformation of the concrete. Measurements were taken at regular intervals during the early curing period to capture the rapid changes in shrinkage behavior. Special care was taken to minimize external disturbances and ensure measurement accuracy throughout the testing duration. The overall technical flowchart is illustrated in Figure 3.

## 3. Test Results and Discussions

### 3.1. Mechanical Properties’ Test Results

**Figure 4:**
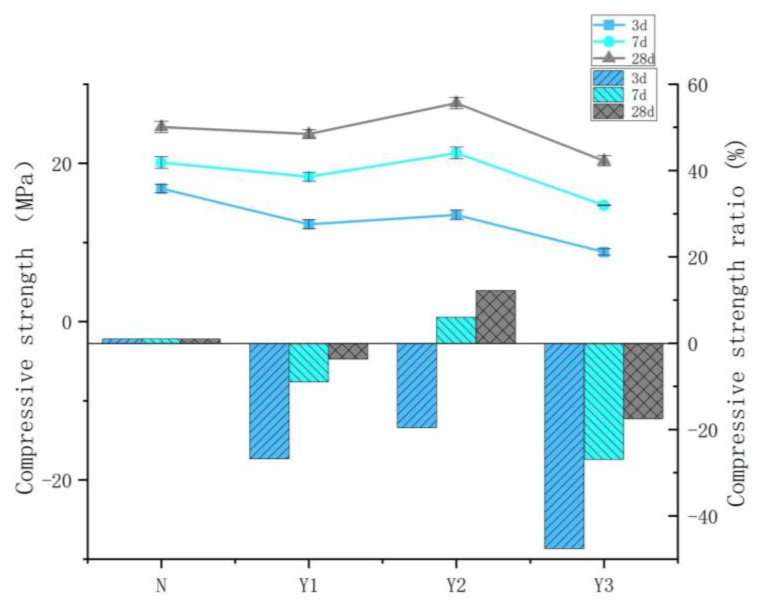
Compressive strength of concrete with different dosages of temperature-control admixture.

The compressive strengths of each group are compared with those of Group N at corresponding curing ages. It is observed that the relative compressive strength values of all three groups decrease over time. Notably, the Y2 group exhibits a positive relative strength value, showing an increasing trend with age. The maximum relative strength of the Y2 group reaches 12.2%. As shown in Figure 4, at the early curing stage (3 days), the relative compressive strength values of groups Y1, Y2, and Y3 are −26.8, −19.6, and −47.6%, respectively. After 7 days of curing, these values increase to −9.0, 6.0, and −26.9%, respectively. By 28 days, the values stabilize at approximately −3.7% for Y1, 12.2% for Y2, and −17.5% for Y3. The results indicate that concrete incorporating the temperature-control admixture exhibits relatively lower compressive strength during the early curing stage. However, by 28 days, this trend has significantly changed. The compressive strength of Group Y1 gradually approaches that of the control group N, while Group Y2 demonstrated superior performance, even exceeding that of the control. Therefore, it can be concluded that the improvement in compressive strength was most pronounced in Group Y2.

**Figure 5:**
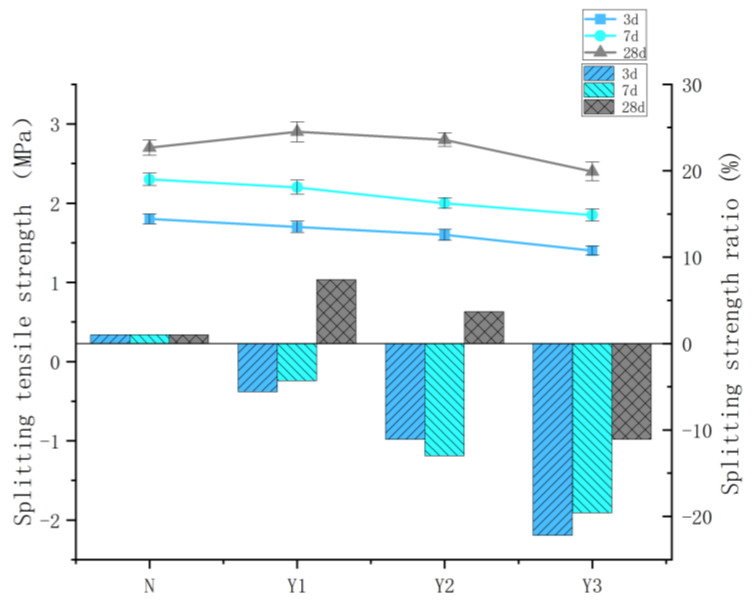
Splitting tensile strength of concrete with different dosages of temperature-control admixture.

The tensile strengths of each group are compared with those of Group N at corresponding curing ages. It is found that the relative tensile strength values of all three groups generally decreased over time. At 28 days, the tensile strength values of Groups Y1 and Y2 surpass that of Group N, with their relative differences being positive integers, indicating a significant improvement in tensile strength, especially for Group Y1. As shown in Figure 5, at the early curing stage (3 days), the relative tensile strength values of groups Y1, Y2, and Y3 are −5.6, −11.1, and −22.2%, respectively. After 7 days of curing, these values increase to −4.3, −13.0, and −19.6%, respectively. By 28 days, the values stabilize at approximately 7.4% for Y1, 3.7% for Y2, and −11.1% for Y3. These results indicate that the incorporation of the temperature-control admixture initially suppressed the development of splitting tensile strength at early ages. However, by 28 days, the tensile strength not only recovers but also shows an enhancement compared to the control concrete. Among all groups, Y1 exhibits the most significant improvement. Although the improvement in splitting tensile strength of the Y2 group is relatively modest with 3.7% at 28 days, it remains meaningful for enhancing the tensile performance of concrete, which is critical in controlling crack initiation and propagation in structural applications.

**Figure 6:**
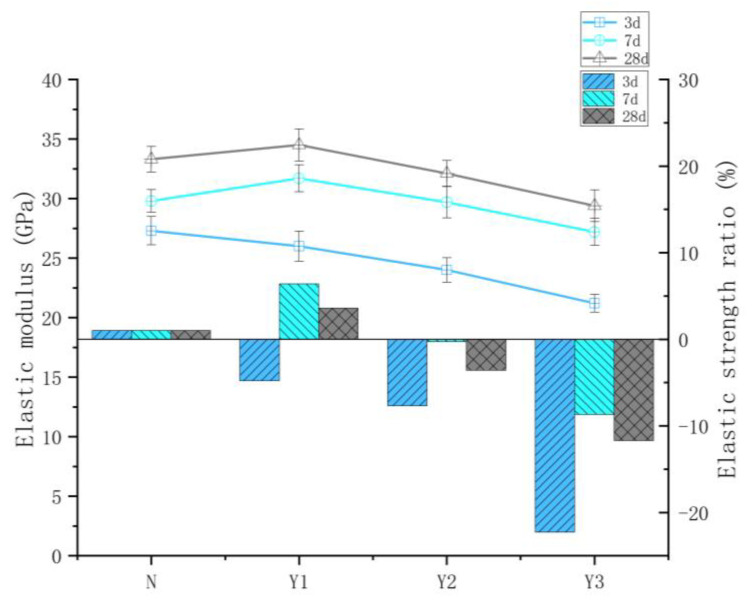
Elastic modulus of concrete with different dosages of temperature-control admixture.

An interesting phenomenon is observed when comparing the elastic modulus of each group with that of Group N at corresponding curing ages. All groups incorporating the temperature-suppressing admixture exhibit a similar trend: the relative elastic modulus initially increases and then decreases over time. This indicates that between 3 and 7 days, the development rate of the elastic modulus in the admixture-containing groups was higher than that of Group N, while from 7 to 28 days, the rate of increase slowed down. Notably, the elastic modulus values of Group Y1 at both 7 and 28 days exceed those of Group N, suggesting a sustained enhancement. As shown in Figure 6, at the early curing stage (3 days), the relative elastic modulus values of groups Y1, Y2, and Y3 are −4.8, −7.7, and −22.3%, respectively. After 7 days of curing, these values increased to 6.4, −0.3, and −8.7%, respectively. By 28 days, they stabilize at approximately 3.6% for Y1, −3.6% for Y2, and −11.7% for Y3. Although the incorporation of the temperature-control admixture may affect the early-age strength development of concrete, with proper mix design and appropriate curing conditions, the long-term mechanical performance can still meet the design requirements. As shown in Figure 6, the Y1 group exhibits the most significant improvement in elastic modulus, demonstrating a positive effect on the long-term strength development of the concrete.

### 3.2. Shrinkage Test Results

Concrete shrinkage behavior primarily includes two components: autogenous shrinkage and drying shrinkage. Autogenous shrinkage arises from chemical shrinkage during cement hydration and self-desiccation, whereas drying shrinkage is closely related to capillary stresses induced by humidity gradients in the external environment. The autogenous shrinkage should be taken into consideration more in concrete with a low water-to-binder ratio (w/b) (for example, high-strength or high-performance concrete) because the autogenous shrinkage of low w/b concrete is much larger than that of ordinary concrete, which may result in cracking at the early ages [48]. Autogenous shrinkage can be neglected when the w/b ratio is higher than 0.42 [49,50]. Moreover, in this study, the fly ash content reached 35%, which substantially reduces the autogenous shrinkage [51]. More notably, early-age autogenous shrinkage in such mixtures may even result in slight expansion, primarily due to the formation of expansive products such as ettringite (AFt) during the initial stages of hydration. This phenomenon has been reported in other studies involving blended cement systems. Based on these considerations, the contribution of autogenous shrinkage is assumed to be minimal in this study. Therefore, in this study, the total measured shrinkage is approximated as drying shrinkage [52].

Concrete shrinkage is most pronounced during the early stages, particularly for Group N. After demolding, the exposure of the specimen surface to air increased, leading to more severe moisture evaporation. By 28 days, Group N exhibits the greatest degree of shrinkage, while groups Y1 and Y2 show similar shrinkage behaviors. Group Y3 exhibits the lowest shrinkage among all groups. The addition of the temperature-control admixture effectively suppresses the internal temperature rise of the concrete, thereby reducing temperature differentials at the same curing age and contributing to a lower shrinkage rate. Furthermore, as the dosage of the temperature-control admixture increases, the shrinkage continuously decreases, indicating that the admixture played a positive role in controlling shrinkage.

**Figure 7:**
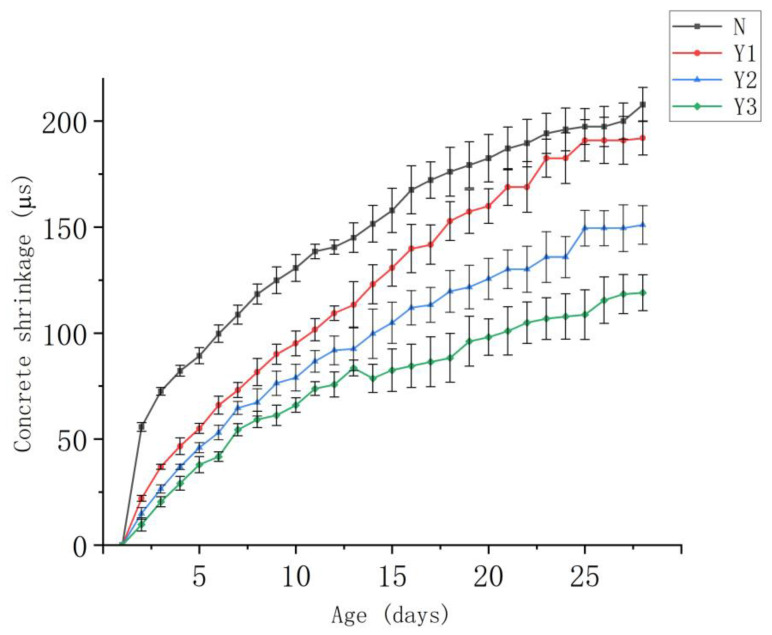
Comparison of concrete shrinkage between different concrete mixes.

Figure 7 shows the evolution of shrinkage over time for all concrete groups after demolding. As observed in Figure 7, at 28 days, the relative shrinkage values for concrete mixtures with 0.05, 0.1, and 0.15% temperature-control admixture are 192, 151, and 119 μs, respectively. These values were all lower than the shrinkage value of the control concrete (207.8 μs) at the same age, corresponding to reductions of 7.6, 27.3, and 42.7%, respectively, compared to the control.

## 4. Modeling of Concrete Shrinkage

Concrete shrinkage is a complex phenomenon influenced by various factors such as material properties, environmental conditions, and curing history. Accurate prediction of shrinkage is critical for the design, serviceability, and long-term durability of concrete structures. Traditional shrinkage models embedded in design codes, such as Eurocode 2, Model Code 2010, AS 3600, and the Bazant B4 model, are primarily developed based on conventional concrete without the incorporation of modern admixtures like temperature-control agents.

However, the addition of temperature-control admixtures alters the internal hydration heat evolution, pore structure development, and moisture distribution, potentially affecting the shrinkage behavior. As a result, existing models may not accurately capture the shrinkage characteristics of such modified concretes. Therefore, there is a pressing need to systematically evaluate the applicability of existing models, develop a more accurate shrinkage prediction model tailored to concrete containing temperature-control admixtures, and validate the proposed model against experimental data.

### 4.1. Review of Shrinkage Models in Existing Codes

Several predictive models have been developed to estimate concrete shrinkage, including those presented in Eurocode 2, Model Code 2010, AS 3600, and the Bazant B4 model. These models were mainly derived from conventional concrete and may not fully capture the behavior of concrete incorporating modern admixtures. A brief review of each model is presented below.

#### 4.1.1. Eurocode 2

Eurocode 2 (EC2) provides simplified empirical models for shrinkage and creep, primarily based on conventional normal-strength concrete. It separates total shrinkage into autogenous and drying components and offers correction factors for environmental influences and member size. EC2 is widely used in Europe for standard construction materials under typical humidity and temperature conditions. However, its applicability is limited for high-performance concrete or concretes modified with advanced admixtures, as it does not account for significant changes in hydration and pore structure development. The shrinkage model in EC2 can be expressed as follows:

(1) $$\varepsilon_{cd}=\frac{\left(t-t_{s}\right)}{(t-t_{s})+0.04\sqrt{{h_{0}}^{3}}}\times k_{h}\times \varepsilon_{cd,0}$$

where $\varepsilon_{cd}$ is the drying shrinkage; $t_{s}$ is the age of the concrete at the beginning of drying shrinkage, and it equals 1 in this study; $h_{0}$ is the notional size of the cross-section, calculated as $h_{0}=2A_{c}/u$, where $A_{c}$ is the cross-sectional area; and u is the perimeter exposed to drying. In this study, the specimen with a cross-section of 100 mm × 100 mm, $h_{0}$ = 50 mm; $k_{h}$ is a coefficient depending on the notional size, and it equals 1 when $h_{0}$ is less than 100 mm; and $\varepsilon_{cd,0}$ is the expected mean values of drying shrinkage values depending on the compressive strength and relative humidity, and it equals 0.48 in this study.

#### 4.1.2. Fib Model Code 2010

Building upon earlier models such as the CEB-FIP Model Code 1990, the fib Model Code 2010 (MC2010) refines shrinkage and creep formulations, particularly addressing the behavior of high-strength concrete. It introduces more detailed environmental sensitivity and time-development functions, offering better adaptability across a wider range of concrete types and exposure conditions. While MC2010 improves predictive accuracy for concretes with supplementary cementitious materials, it still assumes conventional hydration kinetics and does not specifically address the effects of temperature-control admixtures.

(2) $$\varepsilon_{cd}=\left(220+110\times \alpha_{ds1}\right)\times e^{\left(-\alpha_{ds2}\times f_{cm}\right)}\times 10^{-6}\times \beta_{RH}\left(RH\right)\times \sqrt{\frac{t-t_{s}}{0.035\times {h_{0}}^{2}+\left(t-t_{s}\right)}}$$

where $\alpha_{ds1}$ and $\alpha_{ds2}$ are coefficients, depending on the type of cement, with values of 4 and 0.012, respectively; $\beta_{RH}\left(RH\right)$, taking into account the effect of the ambient relative humidity, and it equals $-1.55\times [1-{\left(\frac{RH}{100}\right)}^{3}]$; *h_0_* is the notional size of the cross-section, and it equals 50 mm; and (*t−t_s_*) is the duration of drying in days.

#### 4.1.3. AS 3600:2018

In contrast to the more refined approaches of EC2 and MC2010, AS 3600:2018 adopts a simplified model aimed at practical design applications. Calibrated primarily for conventional concrete used in typical Australian building environments, it emphasizes ease of use over high precision. Although sufficient for routine structural design, AS 3600 offers limited accuracy for modern concretes containing high volumes of supplementary materials or new-generation chemical admixtures, such as temperature-control agents.

(3) $$\varepsilon_{cd}=\frac{0.8+1.2\times e^{\left(-0.005\times h_{0}\right)}\times {\left(t-t_{s}\right)}^{0.8}}{{\left(t-t_{s}\right)}^{0.8}+0.15\times h_{0}}\times k_{4}\times \left(0.9-0.005\times f_{c}\right)\times {\varepsilon^{*}}_{cd.b}$$

where *k_4_* is equal to 0.6 for a temperate inland environment; where ${\varepsilon^{*}}_{cd.b}$ = 8 × 10^−4^ as per AS3600-2018. *h_0_* is the notional size of the cross-section, and it equals 50 mm; and (*t−t_s_*) is the duration of drying in days.

#### 4.1.4. Bazant B4 Model

For more comprehensive analysis, the Bazant B4 model provides a physically based formulation for predicting shrinkage and creep, integrating a wide range of factors such as age, humidity, size effect, and material properties. It achieves high accuracy across various concrete types and exposure conditions and is often used in research and advanced engineering analyses. Despite its robustness, the B4 model requires extensive input parameters and complex calibration, making it less practical for everyday design use. Additionally, it has limited direct calibration for concretes modified with temperature-control admixtures, necessitating careful parameter adjustments. Moreover, the Bazant B4 model also has a simplified model, only considering the compressive strength to evaluate the shrinkage properties of concrete. As such, two forms of the Bazant B4 model, including regular and simplified models, are considered in this study.

(4) $$\varepsilon_{cd}=\varepsilon_{cd,\infty }\times k_{h}\times S(t)$$

(5) $$\varepsilon_{cd,\infty }=-\varepsilon_{0}\times k_{\varepsilon a}\times \frac{E\left(7+600\right)}{E\left({\tilde{t}}_{s}+\tau_{sh}\right)}$$

(6) $$S(t)=\tanh\sqrt{\frac{t}{\tau_{sh}}}$$

(7) $$\tau_{sh}=\tau_{0}\times k_{\tau a}\times {\left(k_{s}\times h_{0}\right)}^{2}$$

where $\varepsilon_{cd,\infty }$ is the shrinkage correction for the effect of aging on elastic stiffness, and $k_{\varepsilon a}$ and $k_{\tau a}$ are the parameters related to aggregate types. In this study, $k_{\varepsilon a}$ and $k_{\tau a}$ are equal to 1.6 and 2.3 as sandstone was used; $k_{s}$ is a parameter depending on specimen geometry, and it selected as 1.25; and $k_{h}$ is humidity dependence factor, which can be determined using $k_{h}=1-h^{3}$ when relative humidity is less than 98%. In this study, $k_{h}$ is calculated as 0.875, and S(t) is the time function which related to drying shrinkage halftime $\tau_{sh}$, as per Equation (7).

Moreover, $\varepsilon_{0}$ and $\tau_{0}$ are parameters related to the mix proportion details such as aggregate-to-binder ratio, water-to-binder ratio, and binder content. These parameters can be calculated using Equations (8) and (9):

(8) $$\varepsilon_{0}=-\varepsilon_{cem}\times {\left(\frac{a/c}{6}\right)}^{p_{\varepsilon a}}\times {\left(\frac{w/c}{0.38}\right)}^{p_{\varepsilon w}}\times {\left(\frac{6.5\times c}{2350}\right)}^{p_{\varepsilon c}}$$

(9) $$\tau_{0}=\tau_{cem}\times {\left(\frac{a/c}{6}\right)}^{p_{\tau a}}\times {\left(\frac{w/c}{0.38}\right)}^{p_{\tau w}}\times {\left(\frac{6.5\times c}{2350}\right)}^{p_{\tau c}}$$

where the parameters such as $\tau_{cem}$, $p_{\tau a}$, $p_{\tau w}$, $p_{\tau c}$, and others depend on the types of cement and their values can be found in Table 4.

On the other hand, in the B4 simplified model, $\varepsilon_{0}$ and $\tau_{0}$ have similar forms such as $\varepsilon_{s,cem}$ and $\tau_{s,cem}$ in terms of mean compressive strength $\bar{f_{c}}$. The expression of $\varepsilon_{s,cem}$ and $\tau_{s,cem}$ is shown as follows:

(10) $$\varepsilon_{0}=\varepsilon_{s,\;cem}(\frac{\bar{f_{c}}}{40}{)}^{S_{\varepsilon f}}$$

(11) $$\tau_{0}=\tau_{s,\;cem}(\frac{\bar{f_{c}}}{40}{)}^{S_{\tau f}}$$

### 4.2. Comparison Between Experimental Data and Model Predictions

**Figure 8:**
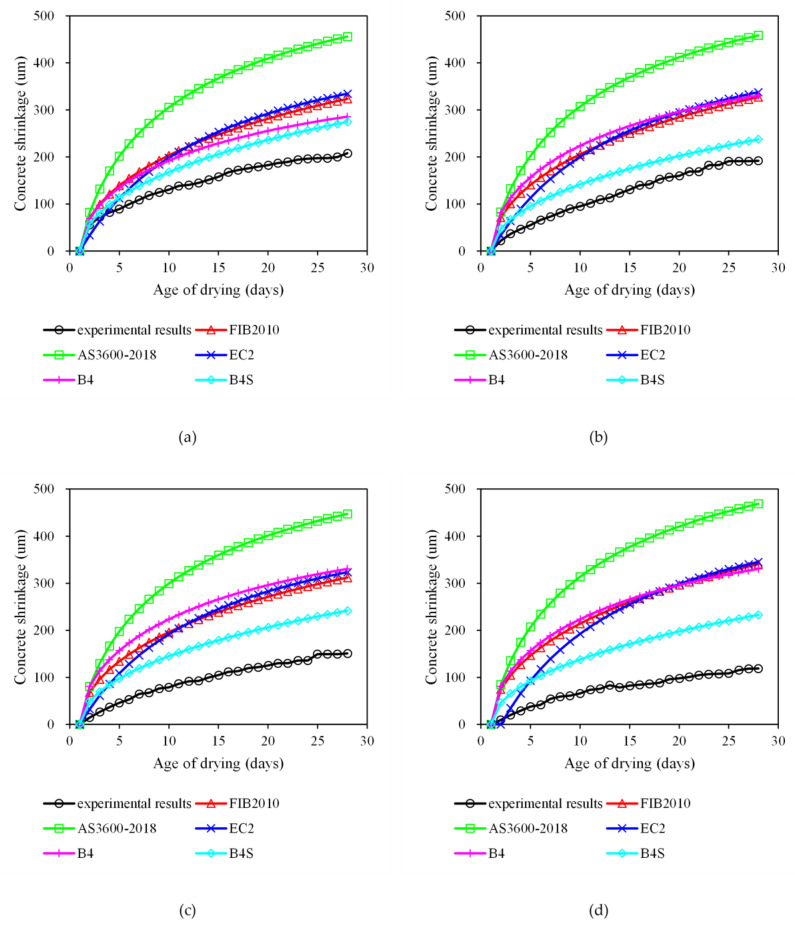
Comparisons between experimental and model results for: (**a**) N; (**b**) Y1; (**c**) Y2; (**d**) Y3.

Figure 8 compares the experimental shrinkage evolution of fly ash concrete (Groups N, Y1, Y2, and Y3) with predictions from five established models: Eurocode 2, fib Model Code 2010, AS 3600-2018, the Bazant B4 model, and its simplified variant (B4S model). It can be observed from Figure 8 that all models consistently overestimated total shrinkage, particularly for TCA-modified concrete. While such conservatism may be beneficial for safety considerations, excessive prediction errors, exceeding 100% in some cases, can lead to unnecessary design conservatism and increased material costs. At 28 days, the experimental shrinkage for Group N (0% TCA) reached 207.8 μm/m, while models predict 274.7–456 μm/m (mean absolute percentage error, MAPE: 32.2–87.2%). This gap widens with increasing TCA dosage (e.g., Y3: experimental 119 μm/m vs. predicted 233.2–468.6 μm/m; MAPE: 96.0–293.8%), indicating that existing formulations fail to capture TCA-induced suppression of moisture loss and pore refinement. B4S exhibits the closest agreement (MAPE: 32.2–96% across groups), attributed to the least consideration of affecting factors of the B4 simplified model. When only compressive strength is considered, the predicted value is the smallest. MC2010 and B4 show moderate accuracy (MAPE: 37.5–186.3%), but underestimate early-age shrinkage (≤7 days) by up to 25% for Y1–Y3, due to omitted TCA retardation effects on capillary stress development. EC2 and AS 3600 perform the poorest (MAPE: 61.0–293.7%), as their empirical humidity corrections neglect TCA’s role in delaying internal RH drop. Model-experiment misalignment peaked during the 3–7 days acceleration phase (e.g., EC2 error > 129% for Y2 at Day 7), coinciding with TCA suppression of temperature rise (Figure 4) and hydration kinetics. This confirms that models calibrated for conventional concrete misrepresent the time-dependent shrinkage evolution of TCA-modified systems, where delays in peak temperature alter drying shrinkage drivers.

### 4.3. Development of a New Shrinkage Prediction Model

Although the fib Model Code 2010 (MC2010) is developed with improvements over earlier empirical models, including refined environmental sensitivity and applicability to high-performance concrete, it consistently overestimates the shrinkage of fly ash concrete containing temperature-control admixtures (TCAs), as revealed by the experimental data in this study. As shown in Figure 8, the predicted shrinkage values by MC2010 exceed the measured values across all mix groups, with particularly large deviations at early ages. This discrepancy arises because MC2010 does not incorporate the effects of TCAs on hydration kinetics and internal moisture transport, which are critical for accurately modeling the shrinkage behavior of such modified concretes.

Specifically, TCAs are known to reduce peak hydration temperatures and delay early-age thermal evolution, which in turn suppresses capillary tension development and slows the rate of moisture loss. These mechanisms significantly lower both the magnitude and rate of shrinkage, particularly within the first 7–14 days. However, MC2010 assumes standard hydration and shrinkage development, leading to accelerated and excessive shrinkage predictions when applied to TCA-modified systems. Furthermore, the current model lacks a mechanism to account for the admixture dosage effect on ultimate shrinkage strain. Therefore, a modified shrinkage prediction model is necessary to better reflect the reduced and delayed shrinkage progression observed in fly ash concrete incorporating temperature-control admixtures.

To develop the new model, two factors are considered in the revised FIB 2010 model. First, a new factor φ capturing the effect of fly ash is introduced. Second, another factor β related to the content of TCA is added in the time-development function of the revised FIB 2010 model, as shown in Equation (12):

(12) $$\varepsilon_{cd}=\varepsilon_{cds0}(f_{cm})\times \beta_{RH}\times {\beta_{ds}}^{\gamma }\times \varphi$$

where φ is the fly ash factor, and since fly ash reduces the shrinkage of concrete [53], the value of φ is set to be 0.7 based on the least-squares method. γ is the factor related to TCA content, and the value γ can be expressed by a linear function using the least-squares method, as follows:

(13) γ=2.4p+0.5

where p is the content of TCA in percentage.

It should be noted that both the fly ash factor φ and the TCA factor γ are introduced as semi-empirical parameters. This approach is consistent with previous studies where empirical correction factors are employed to capture the influence of supplementary cementitious materials or chemical admixtures when a complete mechanistic model is not available [46]. While these factors do not have direct physical units, they reflect the observed effects of fly ash on pore structure refinement and the influence of TCAs on hydration kinetics and moisture transport. These parameters provide a pragmatic way to incorporate the measured behavior into the predictive framework, serving as a basis for future model refinements that may integrate more mechanistic considerations.

### 4.4. Validation of the Proposed Model

To validate the predictive capability of the newly proposed shrinkage model, comparisons were made between the experimental results, the original Model Code 2010 (MC2010) predictions, and the predictions of the modified model developed in Section 4.3.

**Figure 9:**
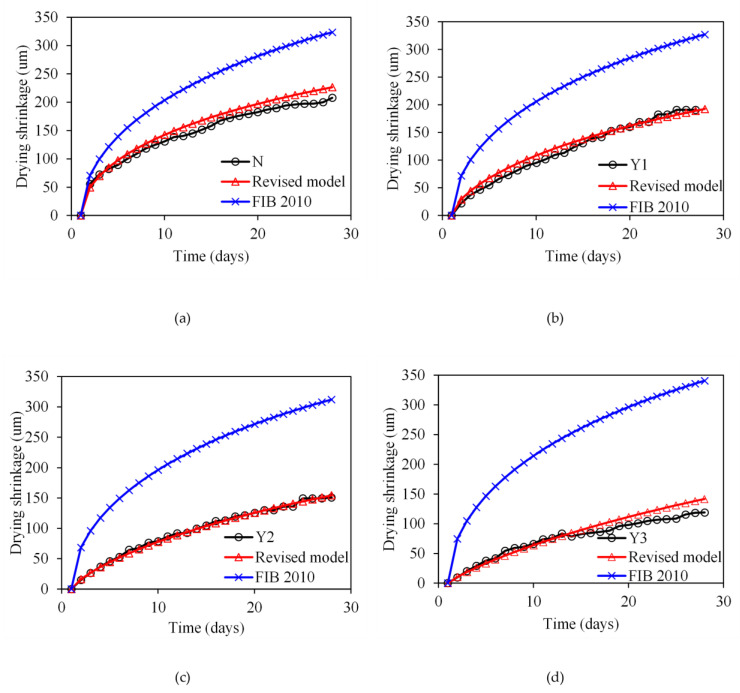
Comparison of experimental shrinkage results with predictions by the original MC2010 model and the proposed modified model for: (**a**) N; (**b**) Y1; (**c**) Y2; (**d**) Y3.

Figure 9 illustrates the comparison of total shrinkage strains over time for different concrete mixtures (N, Y1, Y2, and Y3 groups). It can be observed that the original MC2010 model significantly overestimates the shrinkage strains for all groups, particularly at early ages. This discrepancy can be attributed to the fact that MC2010 does not consider the effects of temperature-control admixtures on hydration heat, internal moisture migration, or pore structure development.

In contrast, the modified model exhibits excellent agreement with the experimental data across all groups. For the control concrete (Group N), the new model accurately captures both the magnitude and the time evolution of shrinkage. For the mixtures incorporating the temperature-control admixture (Groups Y1, Y2, and Y3), the modified model effectively predicts the reduction in ultimate shrinkage strain and the delayed development of shrinkage over time, as observed in the experimental results. These findings demonstrate that the proposed model can reliably predict the total shrinkage behavior of fly ash concrete containing temperature-control admixtures, offering a more accurate tool for structural design and durability assessment. In this study, the parameters recommended by each code were adopted without curve fitting to evaluate the baseline performance of the models. Although curve fitting could reduce the prediction error, it may compromise the general applicability of the models. To improve accuracy, empirical correction factors reflecting TCA dosage and fly ash content were introduced, reducing the mean prediction error to approximately 10%. It should be noted that the proposed model has been validated only against the experimental data from this study. Its general applicability remains to be verified with independent datasets, a broader range of TCA dosages, and other environmental conditions. Therefore, the current formulation should be regarded as a preliminary, empirical tool that offers improved accuracy for the tested system but requires further validation before being applied to diverse concrete compositions. Further studies with broader datasets are required to optimize model parameters and enhance robustness.

**Figure 10:**
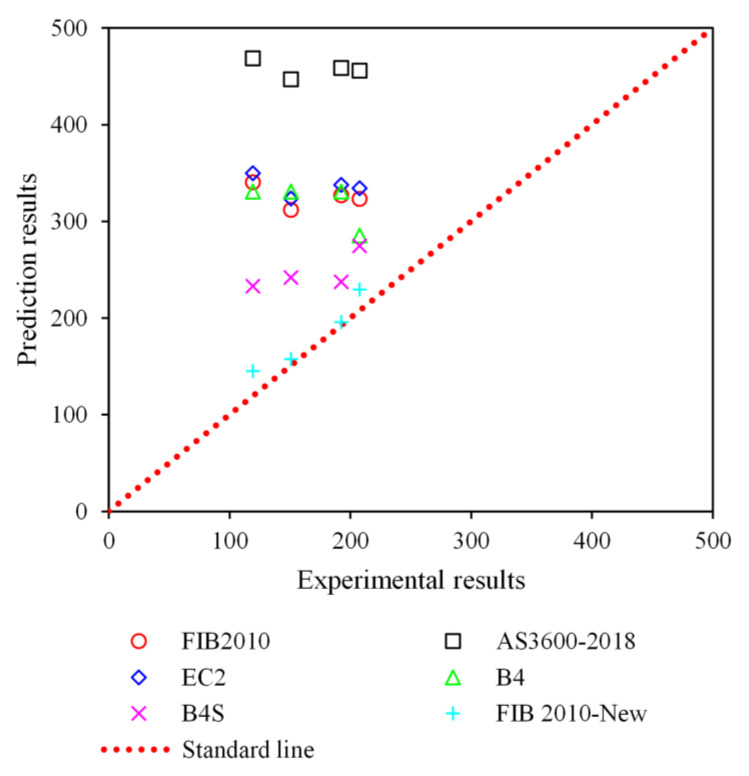
Statistical analysis of results using existing and proposed model.

Figure 10 presents a comparative analysis between experimental shrinkage values and predictions from six models: FIB 2010, AS3600-2018, EC2, B4, B4S, and the proposed FIB 2010-New. Ideally, model predictions should align along the standard 1:1 reference line; however, significant deviations are observed for most models. Notably, AS3600-2018 and EC2 considerably overpredict shrinkage, with average prediction-to-experiment ratios (Mean) of 2.87 and 2.11, respectively, indicating poor compatibility with concrete incorporating temperature-control admixtures. FIB 2010 and B4 also show consistent overestimations (Mean ≈ 2.0), although slightly less severe.

In contrast, the modified FIB 2010-New model exhibits the lowest average error (Mean = 1.10), closely approximating actual shrinkage behavior. Furthermore, it demonstrates superior consistency and reliability, with the lowest standard deviation (Std = 0.09) and coefficient of variation (CoV = 0.08) among all models. This confirms the enhanced predictive accuracy and stability of the proposed model when applied to fly ash concrete modified with temperature-control agents. The improved performance is attributed to its incorporation of admixture-sensitive parameters and revised time-development functions, making it more suitable for modern concrete systems where conventional models fall short.

### 4.5. Clarification and Limitations of the Modified Model

#### 4.5.1. Origin and Interpretation of Coefficients

The proposed model introduces two empirical coefficients, γ and φ, to extend the fib Model Code 2010 framework for shrinkage prediction in fly ash concrete incorporating TCAs. A fixed reduction factor φ = 0.7 is used to account for the known shrinkage-reducing effects of fly ash. This value was obtained by minimizing the mean squared error between experimental and predicted shrinkage for the control group (with 35% FA content), consistent with prior studies reporting 20 to 40% shrinkage reduction due to fly ash [53]. It reflects the influence of reduced hydration heat, delayed pozzolanic activity, and refined pore structure induced by fly ash. A dosage-dependent factor γ = 2.4 × TCA (in percentage) + 0.5 is introduced in the time-development function to capture the observed delay in shrinkage evolution. This coefficient is derived through least-squares regression of the shrinkage development curves for mixtures Y1, Y2, and Y3. The linear formulation reflects the near-linear suppression of internal moisture loss and capillary tension development with increasing TCA dosage.

#### 4.5.2. Validity Domain and Assumptions

The proposed model was developed based on experiments conducted under controlled laboratory conditions and is valid within the boundaries of those conditions. Specifically, the model assumes an ambient temperature of 20 ± 2 °C and relative humidity of 50 ± 5%, with concrete mixtures incorporating 35% fly ash as cement replacement and temperature-control admixture dosages ranging from 0 to 0.15% by binder weight. The w/b was maintained at 0.42. Early-age curing was carried out under sealed conditions to minimize external drying, followed by constant ambient exposure. The model assumes that total shrinkage is primarily governed by drying shrinkage, as the autogenous component is considered negligible in high-volume fly ash systems with moderate w/b ratios. These constraints should be acknowledged when applying the model outside of this validated parameter space.

#### 4.5.3. Limitations of the Proposed Model

Although the proposed model significantly improves shrinkage prediction accuracy for fly ash concrete modified with TCAs, it remains a semi-empirical formulation and, therefore, subject to several limitations. Firstly, it does not explicitly account for changes in ambient humidity or temperature, which can influence drying shrinkage behavior over time. The internal mechanisms of moisture transport, capillary tension development, and pore pressure evolution are not captured in the model’s structure. Secondly, the model parameters were calibrated for a single type of fly ash and TCA product; hence, their applicability to other materials remains uncertain. Additionally, the model was not tested under variable environmental conditions such as outdoor weathering or accelerated drying, nor does it address the interaction of shrinkage with other deformation mechanisms such as creep or restrained cracking. These limitations underscore the need for cautious application and further validation before broader adoption in diverse engineering contexts.

#### 4.5.4. Future Research Directions

To enhance the scientific robustness and predictive capability of the proposed model, future research should focus on integrating more comprehensive datasets and advanced modeling techniques. A promising direction involves the incorporation of multi-variable regression or machine learning approaches trained on large-scale experimental datasets that include various SCMs and TCA types. Further, linking microstructural indicators such as mercury intrusion porosimetry results, SEM images, or X-ray computed tomography with shrinkage behavior could provide deeper insight into the role of admixtures in moisture retention and pore evolution. Another avenue includes the integration of hydration heat measurements and internal relative humidity monitoring to develop a more mechanistic understanding of shrinkage development. Finally, extending model validation to different environmental exposures, concrete mix designs, and structural conditions will be essential to ensure the model’s adaptability to field applications. These enhancements will contribute to a more universal and scientifically grounded shrinkage prediction framework for next-generation concrete systems.

## 5. Conclusions

This study investigated the effects of a temperature-control admixture on the shrinkage behavior and mechanical properties of fly ash concrete. Additionally, the applicability of existing shrinkage prediction models was evaluated, and a modified shrinkage prediction model was proposed and validated. Based on the experimental results and analysis, the following conclusions can be drawn:

- The incorporation of the temperature-control admixture significantly reduced both the early-age and long-term shrinkage of concrete. As the dosage of the admixture increased, the total shrinkage strain progressively decreased. At 28 days, concretes with 0.05, 0.10, and 0.15% admixture content exhibited reductions in shrinkage of 6.4, 9.1, and 24.5%, respectively, compared to the control concrete.
- While the temperature-control admixture slightly suppressed the early-age development of mechanical properties, it did not adversely affect the long-term strength. At 28 days, the compressive strength, splitting tensile strength, and elastic modulus of concrete with appropriate admixture dosages (especially 0.10%) were comparable to or exceeded those of the control concrete.
- Existing shrinkage prediction models, including Eurocode 2, fib Model Code 2010, AS 3600, and the Bazant B4 model, generally underestimated the shrinkage strains of concrete incorporating temperature-control admixtures. This discrepancy highlights the need for model adjustments to accommodate the modified hydration and moisture transport characteristics induced by such admixtures.
- A modified shrinkage prediction model was developed by extending the fib Model Code 2010 through the incorporation of a fly ash–dependent correction factor and a dosage-sensitive time-development function for TCAs. The model was calibrated using experimental data and achieved substantially lower prediction errors than conventional code-based models, particularly for concrete mixtures incorporating TCAs.
- While the model demonstrates reliable performance within the tested parameter range, including a fly ash replacement rate of 35% and TCA dosages up to 0.15%, it remains semi-empirical in nature, and its applicability to other concrete systems or environmental conditions requires further validation. Nevertheless, it provides a scientifically grounded framework for the improved prediction of drying shrinkage in modified concretes and offers practical support for the design and long-term durability assessment of mass concrete structures where shrinkage control is essential.

## Figures and Tables

**Table 1:** Chemical compositions of OPC and FA.

Chemical Oxides	SiO_2_	CaO	Al_2_O_3_	SO_3_	Fe_2_O_3_	K_2_O	MgO	Na_2_O	TiO_2_
OPC	20.31	65.5	4.8	2.1	4.99	0.4	1.3	0.15	0.39
FA	46.59	4.98	38.52	0.66	3.93	0.66	0.96	0.2	1.69

**Table 2:** Technical properties of temperature-control admixture.

Properties	Specification Value
Temperature reduction rate (%)	≥30
Initial temperature rises time difference for 5 °C (h)	≤48
Permeability height (28 days) (mm)	≤100
Compressive strength ratio (28 days) (%)	≥90
Relative durability (%)	≥150

**Table 3:** Mix design of concrete (kg/m^3^).

Sample ID	Cement	Fly Ash	Fine Aggregate	Coarse Aggregate	Water	TCA
N	280	150	675	1175	180	0
Y1	280	150	675	1175	180	0.05%
Y2	280	150	675	1175	180	0.10%
Y3	280	150	675	1175	180	0.15%

**Table 4:** Relative parameters in Bazant B4 model.

Parameters	$\tau_{cem}$	$p_{\tau a}$	$p_{\tau w}$	$p_{\tau c}$	$\varepsilon_{\mathrm{cem}}$	$p_{\varepsilon a}$	$p_{\varepsilon w}$	$p_{\varepsilon c}$
Values	0.016	−0.33	−0.06	−0.10	360 × 10^−6^	−0.80	1.10	0.11

## Data Availability

The original contributions presented in this study are included in the article. Further inquiries can be directed to the corresponding authors.

## Abbreviations

TCA	Temperature-Control Admixture
JX-E	JX-E is commercial TCA from Jinhua Xinsheng (JX) Co., E is the TCA.
OPC	Ordinary Portland Cement
FA	Fly Ash
w/b	Water-to-Binder Ratio
ASTM	American Society for Testing and Materials
GB	Guobiao (Chinese National Standard)
SD	Standard Deviation
AFt	Ettringite
SEM	Scanning Electron Microscopy
MC2010	fib Model Code 2010
EC2	Eurocode 2
AS 3600	Australian Standard for Concrete Structures
B4	B4 Model
